# Assessment of the Decomposition of Oxo- and Biodegradable Packaging Using FTIR Spectroscopy

**DOI:** 10.3390/ma14216449

**Published:** 2021-10-27

**Authors:** Florentyna Markowicz, Agata Szymańska-Pulikowska

**Affiliations:** Institute of Environmental Engineering, Wrocław University of Environmental and Life Sciences, pl. Grunwaldzki 24, 50-363 Wrocław, Poland; florentyna.markowicz@upwr.edu.pl

**Keywords:** biodegradable and oxo-biodegradable packaging, polymers, MSW composting plant, FTIR spectroscopy

## Abstract

The strength and resistance of plastics at the end of their service life can hinder their degradation. The solution to this problem may be materials made of biodegradable and oxo-biodegradable plastics. The aim of this research was to determine the degree and nature of changes in the composition and structure of composted biodegradable and oxo-biodegradable bags. The research involved shopping bags and waste bags available on the Polish market. The composting of the samples was conducted in an industrial composting plant. As a result of the research, only some of the composted samples decomposed. After composting, all samples were analysed using FTIR (Fourier Transformation Infrared) spectroscopy. Carbonyl index and hierarchical cluster analysis method was used to detect similarities between the spectra of the new samples. The analysis of the obtained results showed that FTIR spectroscopy is a method that can be used to confirm the degradation and detect similarities in the structure of the analysed materials. The analysis of spectra obtained with the use of FTIR spectroscopy indicated the presence of compounds that may be a potential source of compost contamination. Plastics with certificates confirming their biodegradability and compostability should be completely biodegradable, i.e., each element used in their production should be biodegradable and safe for the environment.

## 1. Introduction

Plastic materials are widely used in many industries due to their strength, environmental resistance and flexibility. The growing demand for plastics poses the problem of the increasing amount of waste from plastics at the end of their service life [1]. The characteristics of plastics that used to be an advantage, at the end of their service life are the source of decomposition problems [2]. The solution to this problem may be materials made of biodegradable and oxo-biodegradable plastics [3]. Biodegradable materials should decompose under the influence of macro- and microorganisms. Oxo-biodegradable plastics are also biodegradable, but initiating biodegradation requires “abiotic degradation”, e.g., through the use of heat energy or UV radiation [4,5]. Oxo-biodegradable plastics contain additives that are responsible for the initiation of decomposition (prooxidants). The most common additives on the market are d2w^®^ (biodegradable plastic technology) or TDPA^®^ (Totally Degradable Plastic Additives). The rate of biodegradation is influenced by many factors, e.g., chemical character of the polymer, environmental conditions, microbial population activity [6,7]. According to the manufacturers’ assumptions [8,9,10], biodegradable plastics must fulfil designated functions, that is, be (for example) durable and flexible, and at the same time, at the end of their service life, they should be biodegradable in the environment, i.e., in litter, landfill, compost or soil.

Biodegradable and oxo-biodegradable materials are used to produce waste disposal bags and food/shopping bags. Such products are often used to collect bio-waste because of the information provided by producers about “compostability”, “environmental degradability” of their materials. These products are indeed certified in accordance with European standards, such as EN 13432, which stipulates that bags should be 90% biodegradable within six months. Consumers who use them believe they are helping to protect the environment by using waste disposal bags. Together with bio-waste, the bags are sent to a waste treatment plant (e.g., a composting plant). However, packaging should only be allowed for biological treatment if it actually decomposes under composting conditions [11]. Scientific research on the decomposition of plastics is very often performed in conditions significantly different from real ones [12,13]. It happens that in laboratory conditions there are changes in the structure of the material, but its total decomposition is not proven [14,15], or may concern only a part of the polymers, which are its components [16]. In the event of partial decomposition of the packaging, contamination may remain in the environment, e.g., in the form of microplastics. In addition, even small changes in the structure of plastics (such as discoloration or porosity), may indicate that micro-particles and, with them, contaminants in the form of e.g., toxic elements are released into the environment. Therefore, it is necessary to develop the technical standards specifying how to conduct biodegradation tests in the environment, taking into account real conditions (depending on whether the degradation takes place in water, soil or landfill). The results of the tests carried out in this way should make it possible to determine the time and degree of decomposition, depending on environmental conditions [17].

One method that is suitable for the analysis of samples consisting of different materials is Fourier transform infrared spectroscopy (FTIR) [18,19,20,21,22]. This method has been used, among others, for the identification of polymers in marine waste and even those found in animal organisms. In the case of waste materials, FTIR spectroscopy has been used to determine compost maturity, characterise humic substances [23], present in compost and anaerobic decomposing waste, and identify unknown materials present in waste dumped from a landfill site [24]. The conducted research has shown that with the FTIR method it is possible to obtain a lot of information on samples of complex composition [25]. Due to the possibility of conducting research in a relatively simple and non-destructive way, FTIR spectroscopy can also be used to identify plastics and track changes in their composition [26]. Even on the discoloured surface of plastics, it is possible to observe changes confirming the presence of microorganisms and traces of biodegradation [27]. The degree of degradation of plastics can be assessed on the basis of a decrease in the intensity of the bands indicating the presence of C–H bonds, or the appearance of new bands indicating the presence of oxygen connections, such as C=O, C–O, O–H, O–C=O and C=C [28,29,30].

The aim of the research was to determine the degree and nature of changes in the composition and structure of composted (in real conditions) biodegradable and oxo-biodegradable shopping bags and waste bags. The research was carried out using FTIR spectroscopy, which is often used to analyse the structure and degree of decomposition of various materials. The research involved shopping bags and waste bags available on the market, used by consumers to collect the biodegradable fraction of municipal waste, collected selectively for composting processes. Depending on the degree of decomposition of these products, substances included in the plastics, as well as microplastics resulting from the degradation and defragmentation of the film, may be released into the compost to the environment. On the other hand, insufficiently decomposed plastics (larger fragments) will contaminate the compost, preventing its sale and use.

## 2. Materials and Methods

Shopping bags and waste bags generally available in Poland were selected for the research. According to the information provided on the packaging by the producers, the samples were conducted from biodegradable or oxo-biodegradable materials (foil). Some of the samples were partially or completely coloured (in green, brown, orange or black), while the rest were white.

Twelve samples were selected for the research. Table 1 presents information about the analysed samples.

From selected packages, samples were prepared in the form of sheets with an area of approximately 500 cm^2^. Each sheet was sandwiched between two layers of glass fibre mesh with a mesh size of ca 1 × 1 mm (the mesh with a weave of 238 meshes by 5 cm^2^ was used). The sheet closed in the mesh was stapled to prevent the samples from slipping out. Three sets of sheets were prepared for each test: A—new test, not exposed to UV radiation, B—test exposed to UV rays for 20 h, C—test exposed to UV rays for 50 h. The 36 W UV lamp (OSRAM, Munich, Germany) was used for irradiation. The samples were irradiated from a distance of 0.7 m. The irradiation time was supposed to correspond to the irradiation during the storage of waste in the composting plant for 2 or 5 days. Irradiation with UV rays was to initiate the process of decomposition of oxo-biodegradable polymers. The composting of the samples prepared in this way was conducted in an industrial composting plant (Jarocin, Greater Poland Voivodeship, Poland). Initially, composting took place in closed reactors with active aeration, and then on a heap where the compost matured. The entire process took about 5 months. After composting had finished, the sample sheets were removed from the mesh and rinsed thoroughly with distilled water. After washing, a visual assessment of the condition of the samples was carried out. A detailed description of the method of conducting the research is presented in the work from 2019 [31]. All samples were composted at the same time and under the same conditions. Only three out of the 12 samples had completely decomposed, and one was significantly defragmented.

After conducting a visual inspection, all samples were analysed using FTIR spectroscopy. A Nicolet iN10 MX spectrometer (Thermo Scientific, Waltham, MA, USA) equipped with an adapter was used for the samples. The spectra were recorded in the range of 4000–500 cm^−1^, with a resolution of 4 cm^−1^. Interferograms were obtained from 32 scans. Before beginning scanning the samples, the background was irradiated in an empty transparency adapter. The spectra analysis was conducted for all samples from trials A, B and C after the composting process, as well as for new samples, not subjected to composting, constituting the initial spectra (NEW). All laboratory tests were performed in replicates (several fragments of each sample were analysed). Among the spectra obtained, those were selected that did not show any disturbances, caused e.g., by the presence of water. In the case of different coloured samples, scans of parts with different colours were made. In this way, four sets of spectra were obtained for each sample and analysed. Based on the results of the FTIR analysis, the carbonyl index (CI) values were calculated, according to the Equation (1):

CI = Absorbance at 1713 cm^−1^/Absorbance at 1464 cm^−1^ (1)

CI—absorbance ratio of carbonyl and methylene groups. This allows us to determine the amount of carbonyl compounds formed during the photo-oxidation process [32].

The cluster analysis method was used to detect similarities between the spectra of the new samples. This is one of multivariate analyses, useful in cases of large amounts of data (the graph plot of each spectrum consists of 7209 points). Hierarchical cluster analysis permits group observations into clusters. Inside the clusters similar observations can be found, though the clusters differ from each other. Grouping is based on similarities or distance (dissimilarity). The clusters are aggregated according to a decreasing degree of similarity (or increasing degree of dissimilarity) into one single tree-like cluster, called a dendrogram [33].

Grouping of observations (agglomeration) was conducted by the Ward method (the minimum increase of the sum of squares, MISSQ), sometimes called the “minimum variance” method. This method is based on minimising the heterogeneity (variance) in the clusters and finding the greatest possible similarity between the observations. Many studies have demonstrated its accuracy and usefulness in recreating the original structure of clusters [34,35]. The distances between the objects were determined on the basis of the Euclidean distance.

## 3. Results

All of the samples selected for research were composted under identical conditions in an industrial composting plant. This was to reflect the actual conditions in which the waste bags are sent together with biodegradable waste for processing and to check whether their decomposition is possible during the processing of biodegradable waste.

**Figure 1:**
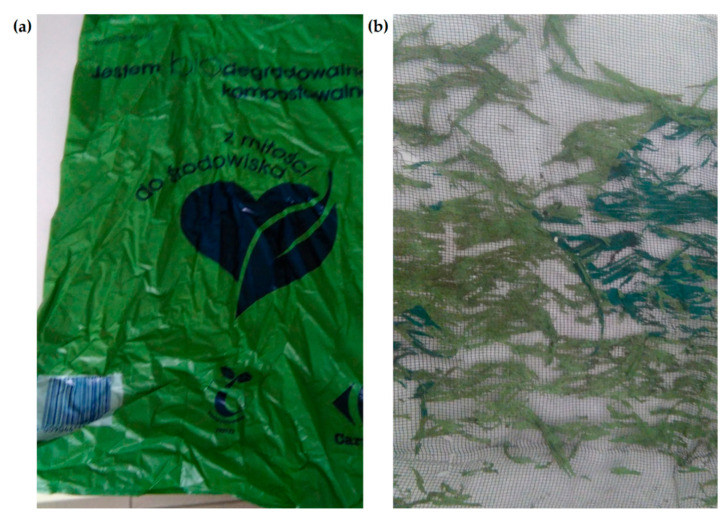
Appearance of sample no. 3: before (**a**) and after the composting process (**b**) [photo F. Markowicz].

Only three out of the 12 samples failed completely. These were samples 1, 2 and 11. They were certified in accordance with the EN 13432 norm, and as described by the producers, they were biodegradable and compostable. Sample no. 3 was significantly defragmented. Figure 1 shows the appearance of the sample before (a) and after the composting process (b). Thin threads remained between the two layers of the sample protection mesh, disintegrating into dust when touched, which made it impossible to make scans. As sample no. 3 also had a compostability certificate, it was considered biodegradable. According to the information provided by the manufacturer, the decomposition of the packaging in sample no. 3 should take place within 6 weeks to a year. Such a long time may not ensure complete decomposition of the material in an industrial composting plant, where the process usually takes several months.

**Figure 2:**
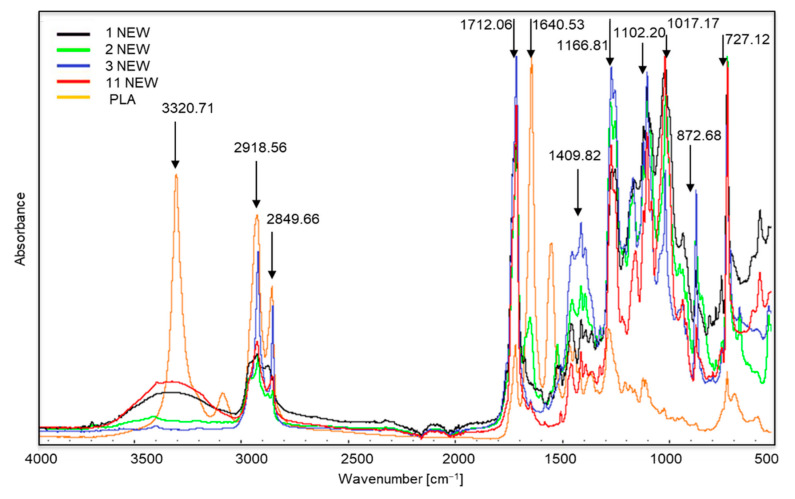
Spectra of new samples 1, 2, 3 and 11, which were decomposed in the composting process. The polylactide (PLA) spectrum was added for comparison.

Figure 2 shows the FTIR spectra of new samples 1, 2, 3 and 11, which were decomposed in the composting process. This was confirmed during a visual assessment. The greatest changes in peak intensity are visible in new sample no. 3. This sample was made from the starch or sweet corn and potatoes, but it was also one of the most intensely coloured. Its composition should have been similar to that in samples 1, 2 and 11, but it may have been made more difficult by the use of a large amount of dyes. Our 2019 research [36], in which we analysed the composition of the tested samples, indicated that sample no. 3 contained large amounts of copper, which is used in the production process to make green dyes.

The spectra of samples 1, 2, 3 and 11 (especially samples 2 and 11) are very similar to the spectra of biodegradable polymers, such as polylactide (PLA) [37]. We can observe characteristic peaks at approximately 3320 cm^−1^, which corresponds to the OH bond, sharp peaks at approximately 1712 cm^−1^ are stretching vibrations of the C≡O ester groups C≡O [38]. Such changes may indicate the use of renewable raw materials in their production, such as corn meal. According to the information provided by the producers, samples 1, 2, 3 and 11 were made entirely of renewable, biodegradable materials.

Table 2 show the changes observed during the analysis of FTIR spectra of samples that did not decompose during composting.

In all samples that did not decompose during composting, was found the occurrence of C–O oxygen groups in carboxylic and ester bonds, C–H bending vibrations and C–Cl (or C–Br) stretching vibrations of alkyl halides. At wavenumbers above 3000 cm^−1^ they occurred stretching vibrations of the N–H amino group (samples no. 7, 8, 9, 12). The bending vibrations of the N–H amino group also occurred with wavenumbers between 1001 a 2000 cm^−1^ (samples no. 4, 7, 8, 9, 12). Moreover, in samples 4 and 6, vibrations of C=C bonds were found, and in sample 6 also vibrations of N–O nitro compounds. The starting decomposition of samples was mainly evidenced by visible discoloration, roughness and lower elasticity of the material.

**Figure 3:**
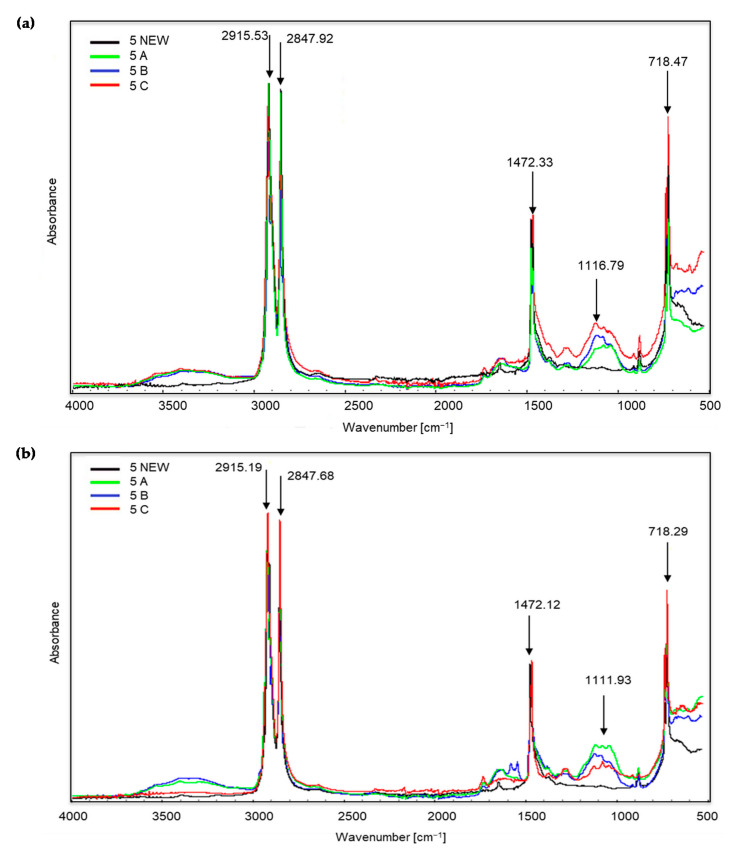
Sample no. 5 spectra: (**a**) spectra generated by the white part of the sample material, (**b**) spectra generated by the coloured part of the sample material.

**Figure 4:**
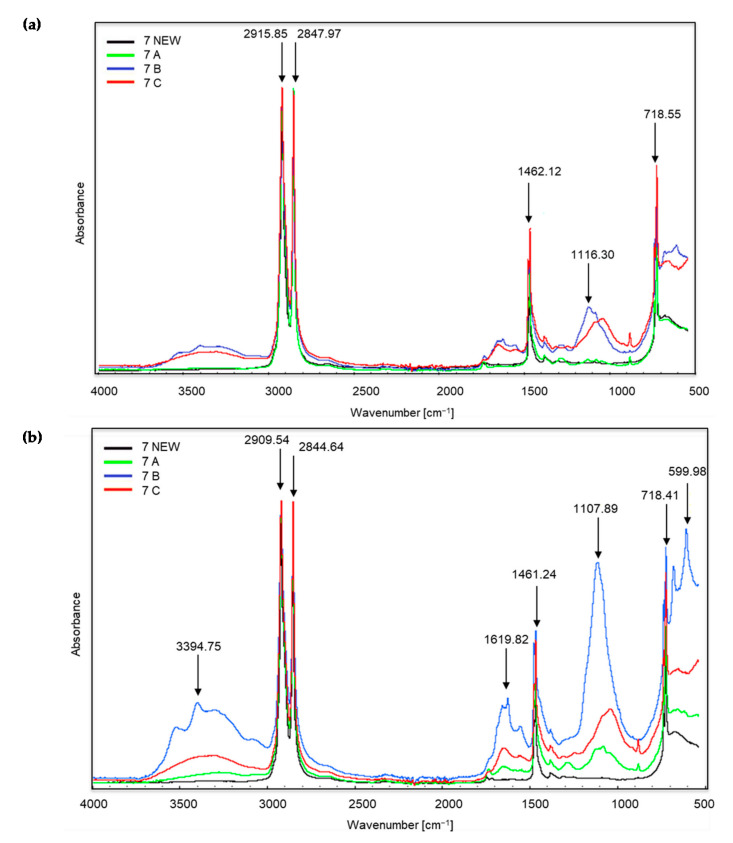
Sample no. 7 spectra: (**a**) spectra generated by the white part of the sample material, (**b**) spectra generated by the coloured part of the sample material.

**Figure 5:**
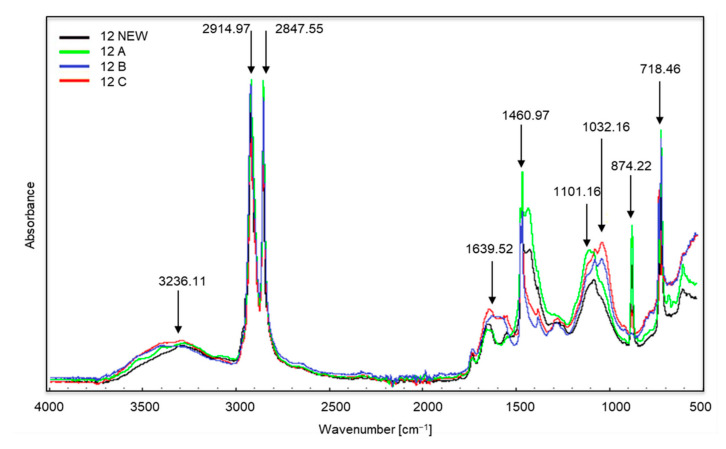
Spectra of sample no. 12 (the entire sample was made up of brown material).

Figure 3, Figure 4 and Figure 5 show the spectra of new samples and three composted variants (Figure 3—sample no. 5, with the addition of TDPA; Figure 4—sample no. 7, with the addition of d2w^®^; Figure 5—the sample described by the manufacturer as a bag for organic waste—no. 12), that did not decompose. Figures showing the FTIR spectra of the remaining samples are included in the supplementary materials. The spectra were obtained during the X-raying of new samples (NEW), composted without irradiation (A), exposed to UV radiation for 20 h and composted (B) and UV irradiated for 50 h and composted (C). The Table of Characteristic IR Absorptions, published by the University of Colorado in Boulder, Department of Chemistry and Biochemistry, was used to analyse the spectra [39,40].

Figure 3 shows the spectra obtained during the X-ray of sample no. 5 (new and three composted variants). The bands visible at the wave numbers of approximately 2915 and 2847 cm^−1^ correspond to the stretching vibrations of the methylene C–H group. Initially, C–H stretching occurs, and then there is a marked drop in the peak, corresponding to bond cleavage. This applies to all of the samples, including the new ones. However, in the case of the coloured part of the material, the peaks are most intense for sample C, i.e., irradiated for 50 h and then composted. Further changes occur only at approximately 1472 cm^−1^ (occurrence of C–H bending vibrations). The presence of the bands in the wave number range 1320–1000 cm^−1^ is small. The growth of the bands around 1116 and 1111 cm^−1^ confirms the appearance of oxygen groups C–O in carboxylic and ester bonds. The bands at approximately 874, 730 and 718 cm^−1^ correspond to the C–Cl stretching vibrations. During visual inspection, no significant changes were noted for sample no. 5 after the composting process.

In the case of sample no. 7 (Figure 4), the first significant changes appeared already in the range of wave numbers amounting to approximately 3394 cm^−1^, which proves the occurrence of stretching vibrations of the amino group N–H. These changes are most intense for the coloured part of sample B, i.e., irradiated for 20 h and then composted. One can also see very intense peaks at approximately 2915, 2909, 2844 and 2847 cm^−1^, which correspond to the vibrations of the C–H methylene group. Initially, C–H stretching occurs, followed by bond cleavage. This applies to all the samples, including the new ones. Both in the white and coloured parts of the material, peaks in this range are the most intense for trial C, i.e., irradiated for 50 h and then composted. Further changes occur in the spectra of coloured materials at the wave numbers of approximately 1619 cm^−1^, which corresponds to the appearance of bending vibrations of the N–H amino group. The peaks at wave numbers around 1461 and 1462 cm^−1^ (for white and coloured material) are the reappearance of C–H bonds (bending vibrations). The next changes concern the wave numbers in the range from approximately 1116 and 1107 cm^−1^ and confirm the appearance of C–O oxygen groups of carboxylic and ester bonds. The bands at approximately 718 cm^−1^ are C–Cl stretching vibrations. Finally, in the coloured part of sample no. 7B, a very intense band appears at a wave number of approximately 599 cm^−1^. According to the authors of the tables [39,40] these are stretching vibrations of C–Br bonds, alkyl halides, which are very stable compounds. The presented changes can be related to the visual assessment. After the composting process, we observed clear changes in the coloured part of sample no. 7. The sample material was also less flexible.

In the case of sample no. 12 (Figure 5) the first changes appear in all samples in the range of wave numbers amounting to approximately 3236 cm^−1^, and this is the occurrence of stretching vibrations of the amino group N–H. The bands visible at the wave numbers of approximately 2914 and 2847 cm^−1^ correspond to the vibrations of the methylene C–H group (stretching and bond cleavage). Changes at approximately 1639 cm^−1^ correspond to the appearance of bending vibrations of the N–H amino group. Further changes occur at approximately 1460 cm^−1^ (occurrence of C–H bending vibrations). The growth of the bands around 1101 and 1032 cm^−1^ is the appearance of oxygen groups C–O in carboxylic and ester bonds. The bands at approximately 874 and 718 cm^−1^ are C–Cl stretching vibrations. Sample no. 12 was made entirely of a brown-coloured material which did not change its structure after the composting process, and the colour changes were minor. The material was still flexible and durable. The producers suggested choosing this type of bag for collecting organic waste (as indicated by the description on the packaging), but even a visual assessment aroused doubts regarding its purpose. The structure, appearance and flexibility of the bag were almost identical to those made of polyethylene.

**Figure 6:**
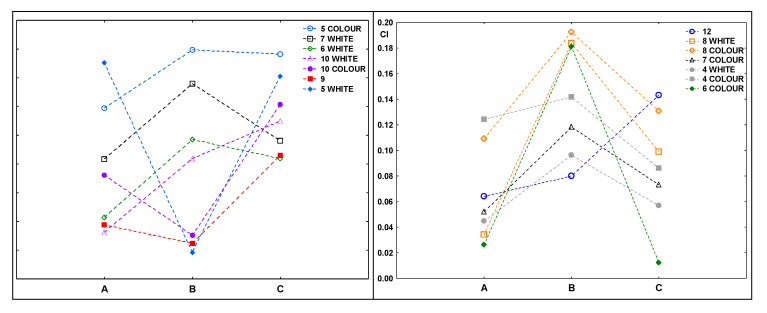
Values of the carbonyl indexes for samples after composting: A—composted without irradiation, B—exposed to UV radiation for 20 h and composted, C—irradiated for 50 h and composted.

Figure 6 shows the carbonyl index values for the samples after composting. In most cases, the indexes achieved higher values for samples that were exposed to UV radiation for 20 h before composting (B).

This does not confirm the results of the spectra analysis, which showed some changes (indicating the ongoing degradation process) in some of the samples irradiated for 50 h. The changes were not clear, they indicated that the decomposition of the samples was starting, even though the composting process was finished.

The use of hierarchical cluster analysis (supplementary materials, Figure S6) confirmed the existence of similar relationships between the spectra of the tested samples. Samples that were completely decomposed during composting (1, 2, 3, 11) ended up in the first main cluster (A), along with the color part of sample no. 6. According to the manufacturer’s declaration, 85% of it was made from sugar cane and there were signs of a biodegradation process. Samples 1, 2, 3 and 11 could be made of materials containing similar components. The remaining samples were included in the second cluster (B). It includes all the samples that were not decomposed in the composting process (except for the coloured part of sample no. 6). This cluster is divided into two smaller ones: the first one (B′) is dominated by coloured parts of samples (4, 10, 5, 8) and samples of uniform colour (9 and 12). It also includes the white parts of samples 4 and 5. Most of the mentioned samples showed an increase in the CI value after 20 h irradiation compared to the samples composted without irradiation. The second cluster (B″) has the most white samples (6, 8, 7, 10), and the coloured part of sample number 7 belongs to this.

## 4. Discussion

Synthetic polymer packaging is readily available, flexible and resistant to many factors. However, they are not renewable, non-biodegradable and pose a threat to the environment. Packages made of natural raw materials (biodegradable and ecological) are usually very light, thin and have low durability. They are not resistant to factors such as water, air and high temperature. It is very difficult to obtain a material that is both biodegradable, environmentally safe and durable [41]. Work on finding the golden mean in this area is ongoing. New types of material are appearing that may replace those previously used. There are many different packages available on the market for consumers described by producers as biodegradable and oxo-biodegradable. The information on these packages is not always accurate. Our research has shown that not all packaging decomposes, despite the fact that the research conditions corresponded to real-world conditions. Another scenario, e.g., longer exposure to UV rays, additional mechanical actions, heating, are not possible or available under real composting conditions.

All samples analysed with the use of FTIR spectroscopy were characterised by differences in the obtained spectra. The samples that did not decompose in the composting process were characterised by intense bands in the 3000–2800 cm^−1^ range, which correspond to the stretching and cleavage of the C–H group bonds. These bands are characteristic, for example, for polypropylene [42], they did not occur in samples 1, 2, 3 and 11, which completely decomposed in the composting process. All of the samples also had new peaks below 1500 cm^−1^, known as the dactyloscopic range. Bands of stretching vibrations of single bonds appear (for example, C–H, C–Cl), corresponding to deformation vibrations, as well as groups of C–O oxygen carboxylic and ester bonds. The observed changes in the spectra indicate the occurrence of the oxidation and degradation process of the materials used in the samples. The spectra of samples 4, 7, 8 and 9, belonging to the films described as oxo-biodegradable, are similar to those presented by Benitez et al., who studied the degradation of polyethylenes with pro-oxidants added. The authors also found that these types of material oxidize the components and degrade the material [43,44]. Also in the case of sample no. 6, some changes appeared, indicating the occurrence of the sample decomposition process (especially the coloured part). In the case of samples no. 7 and 8, the analysis of the spectra revealed the presence of compounds from the group of alkyl halides (C–Br bonds). These compounds are used in the chemical industry and can pose a threat to the environment. Bromine is used in the production of dyes, and its compounds are persistent and stable in the environment. Some of the dyes can also be carcinogenic and mutagenic [45]. Perhaps due to the intense colour of the samples (prints, inscriptions), this compound appeared in them.

Registered changes were found for both white and coloured material. However, they prove abiotic degradation, i.e., the process of initial oxidation of the sample material. This is the stage before proper biodegradation with the use of microorganisms [46,47,48]. It should be noted that the results relate to samples that have already been treated in the composting process. Therefore, they cannot be considered biodegradable, nor can they be considered to degrade in the environment or in compost. In this case, the analysis of spectra obtained using FTIR spectroscopy cannot be treated as the only confirmation of biodegradability. They should be supplemented with other types of test. The carbonyl index allows us to determine the amount of carbonyl compounds formed during the photo-oxidation process. However, it has been shown that a significant part of the oxidation product is omitted, because it evaporates into the atmosphere. Therefore, this indicator cannot be considered as a reliable probe to measure the extent of oxidation and does not reflect the total degradation of the mechanical properties of the polymer [49]. Moreover, the conditions under which biodegradability was tested are important. If these were laboratory conditions, they also do not have to demonstrate the ability to be completely biodegradable, because such conditions are different from real ones and this information may mislead the consumer.

The conducted tests were aimed at assessing the degree and nature of changes occurring during the decomposition of selected packaging and confirming that at various stages of material biodegradation, harmful microplastics and substances contained in plastics may be released into the compost. The processing of plastics together with organic waste causes the pollution to enter the compost, which can then be used as fertiliser [50].

Analysis of the spectra also showed that earlier irradiation of samples did not bring the expected results. According to some authors, earlier exposure of plastics to UV radiation accelerates biodegradation [51] or increases growth of carbonyl indicators [52]. The results show only partial degradation of the samples. In samples no. 5, 6 and 7 there were greater differences between the spectra of samples irradiated for 50 h and the others. Visual inspection confirmed that the structure of some samples had been disturbed. An earlier publication presented the content of selected components in samples before and after the composting process [36]. Analyses have shown that elements such as zinc, copper, chromium and lead can get into the compost. During the decomposition of biodegradable and oxo-biodegradable plastics, microplastic particles may also form, which accumulate in the environment. This was also confirmed by other authors [53,54]. Microplastics are a potential threat to flora and fauna [55], and they affect the functions of soil and microbial communities [56]. The biodegradation of packaging used as waste bags or for storing food is very important, because it concerns many elements of the environment. Errors resulting from a too rash assessment of biodegradability potential may pose a threat to the environment and even to the health and life of animals.

## 5. Conclusions

The analysis of the results of the conducted research allowed for the formulation of the following conclusions:

- As a result of the research, only some of the composted samples decomposed. In the remaining samples, only slight traces of degradation were visible, despite the fact that all the bags selected for the tests were to undergo oxo- and biodegradation.
- FTIR spectroscopy is a method that can be used to confirm the degradation of biodegradable and oxo-biodegradable materials; however, the results obtained in this way may not give unequivocal information about the degree of actual degradation of a given material. It may, however, help in detecting similarities in the structure of the analysed plastics.
- The analysis of spectra obtained with the use of FTIR spectroscopy indicated the presence of compounds in the tested samples that may be a potential source of compost contamination. Apart from the observation of the compounds proving the degradation of the material (oxygen groups, changes in C–H bonds), groups of alkyl halides were detected.
- Alkyl halide groups were found in most of the samples that were not decomposed in the composting process. These were the following samples: oxo-biodegradable (4, 7, 8, 10, 12) and biodegradable (6).
- The analysis of the spectra of the samples subjected to irradiation with UV rays and non-irradiated ones shows that there are no clear differences between the spectra of the irradiated and non-irradiated samples.
- Plastics with certificates confirming their biodegradability and compostability should be completely biodegradable in real conditions (e.g., during composting). Each component used in their production should be similarly tested, i.e., the dyes used should be completely biodegradable and safe for the environment. An example is the intensely coloured biodegradable sample no. 3, the decomposition of which was difficult, and metals such as copper, zinc and chromium could have entered the environment along with the compost.

## Figures and Tables

**Table 1:** The most important information about the samples selected for research.

Sample Number	Type of Polymer	Additional Manufacturer Information
1	Biodegradable, compostable	There is no need to remove from the stream of bio-waste at the composting industry
2	Biodegradable, compostable	It is degraded in composting conditions
3	Biodegradable, compostable	Made on the basis of corn and potato starch
4	Oxo-biodegradable, d2w^®^ additive	It is decomposed under the influence of oxygen, UV and heat, use within 18 months
5	Oxo-biodegradable, TDPA additive	It is subject to accelerated decomposition
6	Biodegradable, LDPE and sugar cane	Sugar cane content above 85%
7	Oxo-biodegradable, d2w^®^ additive	The bag is 100% biodegradable
8	Oxo-biodegradable, HDPE	The bag is oxo-biodegradable by 100%
9	Oxo-biodegradable, d2w^®^ additive	It has the Oxo-biodegradable Plastics Association mark
10	Oxo-biodegradable, HDPE	The bag is oxo-biodegradable by 100%
11	Biodegradable, compostable	Bags for biodegradable waste
12	Oxo-biodegradable, HDPE	Bags for organic waste

**Table 2:** List of changes noticed during the analysis of the Fourier transform infrared (FTIR) spectra of the tested packages (samples 4, 5, 6, 7, 8, 9, 10, and 12).

Sample Number	Wave Number (cm^−1^)			
	>3001	2001–3000	1001–2000	500–1000
4	~3289.77 and 3298.11 (C=C double bonds)	~2915 and 2848 (stretching vibrations of the methylene C–H group)	~1618–1625 (deformation vibrations of the amino group N–H), ~1471 and 1461 (C–H bending vibrations), ~1320–1000 (C–O oxygen groups in carboxylic and ester bonds)	~874, 730 and 718 (C–Cl stretching vibrations of alkyl halides)
5		~2915 and 2847 (stretching vibrations of the C–H methylene group)	~1472 (C–H bending vibrations), ~1116 and 1111 (oxygen groups C–O in carboxylic and ester bonds)	~874, 730 and 718 (C–Cl stretching vibrations of alkyl halides)
6		~2915 and 2847 (stretching vibrations of the C–H methylene group)	~1651 (stretching vibrations of the C=C bonds), ~1525 (N–O nitro compounds), ~1461 and 1472 (C–H bending vibrations), ~1279, 1180 and 1068 (C–O oxygen groups of carboxylic and ester bonds)	~874, 841 and 718 (C–Cl stretching vibrations of alkyl halides)
7	~3394 (stretching vibrations of the amino group N–H)	~2915, 2909, 2844 and 2847 (vibrations of the C–H methylene group)	~1619 (bending vibrations of the N–H amino group), ~1461 and 1462 (C–H bending vibrations), ~1116 and 1107 (C–O oxygen groups of carboxylic and ester bonds)	~718 (C–Cl stretching vibrations), ~599 (stretching vibrations of alkyl halides C-Br bonds)
8	~3266 and 3394 (stretching vibration of the N–H amino group)	~2915 and 2847 (vibrations of the methylene C–H group—bond stretching and cleavage)	~1652 and 1648 (N–H amino group (bending vibrations), ~1461 and 1471 (C–H bending vibrations), ~1279, 1099 and 1077 (C–O oxygen groups in carboxylic and ester bonds)	~874, 844, 730 and 718 (C–Cl stretching vibrations), ~600 (C-Br stretching vibrations)
9	~3266 (stretching vibrations of the amino group N–H)	~2916 and 2848 (vibrations of the methylene C–H group—bond stretching and cleavage)	~1651 (bending vibrations of the N–H amino group), ~1461 (C–H bending vibrations), ~1075 (C–O oxygen groups in carboxylic and ester bonds)	~718 (C–Cl stretching vibrations), ~617 (stretching, broad and strong vibrations of the C–H and –C≡C–H alkynes groups)
10		~2915 and 2847 (vibrations of the C–H methylene group)	~1472 and 1461 (C–H bending vibrations), ~1094 and 1075 (C–O oxygen groups in carboxylic and ester bonds)	~718 (C–Cl stretching vibrations of alkyl halides)
12	~3236 (stretching vibrations of the N–H amino group)	~2914 and 2847 (vibrations of the C–H methylene group—stretching and bond cleavage)	~1639 (bending vibrations of the N–H amino group), ~1460 (C–H bending vibrations), ~1101 and 1032 (C–O oxygen groups in carboxylic and ester bonds)	~874 and 718 (C–Cl stretching vibrations of alkyl halides)

## Data Availability

The data presented in this study are available on request from the corresponding author.

## Supplementary Materials

The following are available online at https://www.mdpi.com/article/10.3390/ma14216449/s1, Figure S1: Sample no. 4 spectra: (a) spectra generated by the white part of the sample material, (b) spectra generated by the coloured part of the sample material, Figure S2: Sample no. 6 spectra: (a) spectra generated by the white part of the sample material, (b) spectra generated by the coloured part of the sample material, Figure S3: Sample no. 8 spectra: (a) spectra generated by the white part of the sample material, (b) spectra generated by the coloured part of the sample material, Figure S4: Spectra of sample no. 9 (the entire sample was made up of white material), Figure S5: Sample no. 10 spectra: (a) spectra generated by the white part of the sample material, (b) spectra generated by the coloured part of the sample material, Figure S6: Dendrogram showing the analyzed new samples. Agglomeration was carried out using the Ward method.
