# Supplementary material for: Assessment of the Decomposition of Oxo- and Biodegradable Packaging Using FTIR Spectroscopy
Source: Materials (Basel). 2021 Oct 27;14(21):6449. doi: 10.3390/ma14216449 (PMC8585154; doi:10.3390/ma14216449)
Supplement: Supplementary file 1 [file materials-14-06449-s001.zip › materials-1396243-supplementary.pdf]

materials

MDPI

Supplementary Materials

Assessment of the Decomposition of Oxo- and Biodegradable Packaging Using FTIR Spectroscopy

Florentyna Markowicz and Agata Szymańska-Pulikowska *

Institute of Environmental Engineering, Wrocław University of Environmental and Life Sciences, pl. Grunwaldzki 24, 50-363 Wrocław, Poland; florentyna.markowicz@upwr.edu.pl
* Correspondence: agata.szymanska-pulikowska@upwr.edu.pl

a) Graphs of the spectra of the analyzed samples no 4, 6, 8, 9 and 10 (Figures S1–S5),

b) Dendrogram showing the similarities between the spectra of the new samples (Figure S6).

Citation: Markowicz, F.; Szymańska-Pulikowska, A. Assessment of the Decomposition of Oxo- and Biodegradable Packaging Using FTIR Spectroscopy. *Materials* **2021**, *14*, 6449. https://doi.org/10.3390/ma14216449

Academic Editor: Loic Hilliou

Received: 10 September 2021
Accepted: 21 October 2021
Published: 27 October 2021

Publisher's Note: MDPI stays neutral with regard to jurisdictional claims in published maps and institutional affiliations.

Copyright: © 2021 by the authors. Licensee MDPI, Basel, Switzerland. This article is an open access article distributed under the terms and conditions of the Creative Commons Attribution (CC BY) license (http://creativecommons.org/licenses/by/4.0/).

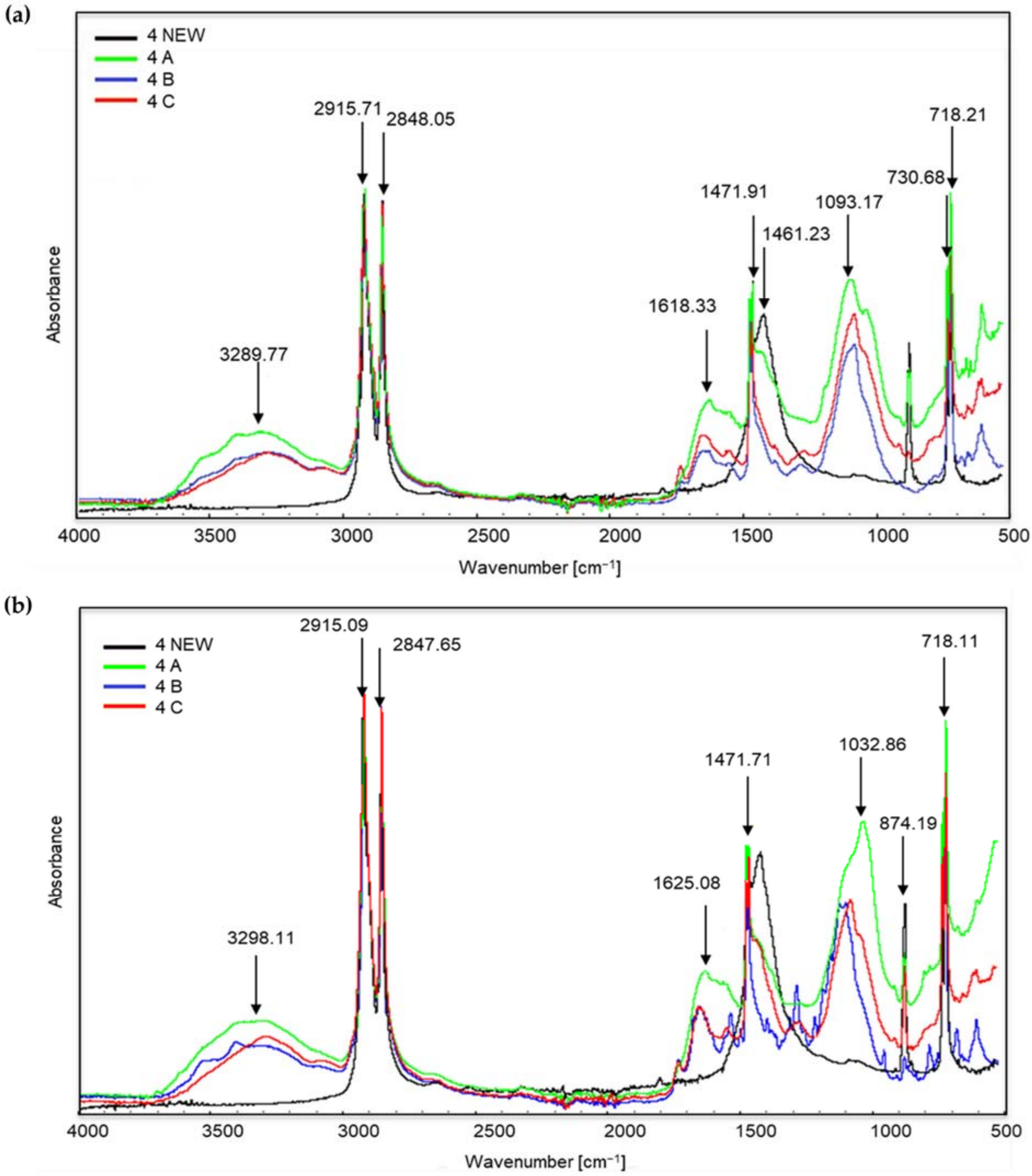

Figure S1. Sample no. 4 spectra: **(a)** spectra generated by the white part of the sample material, **(b)** spectra generated by the coloured part of the sample material.

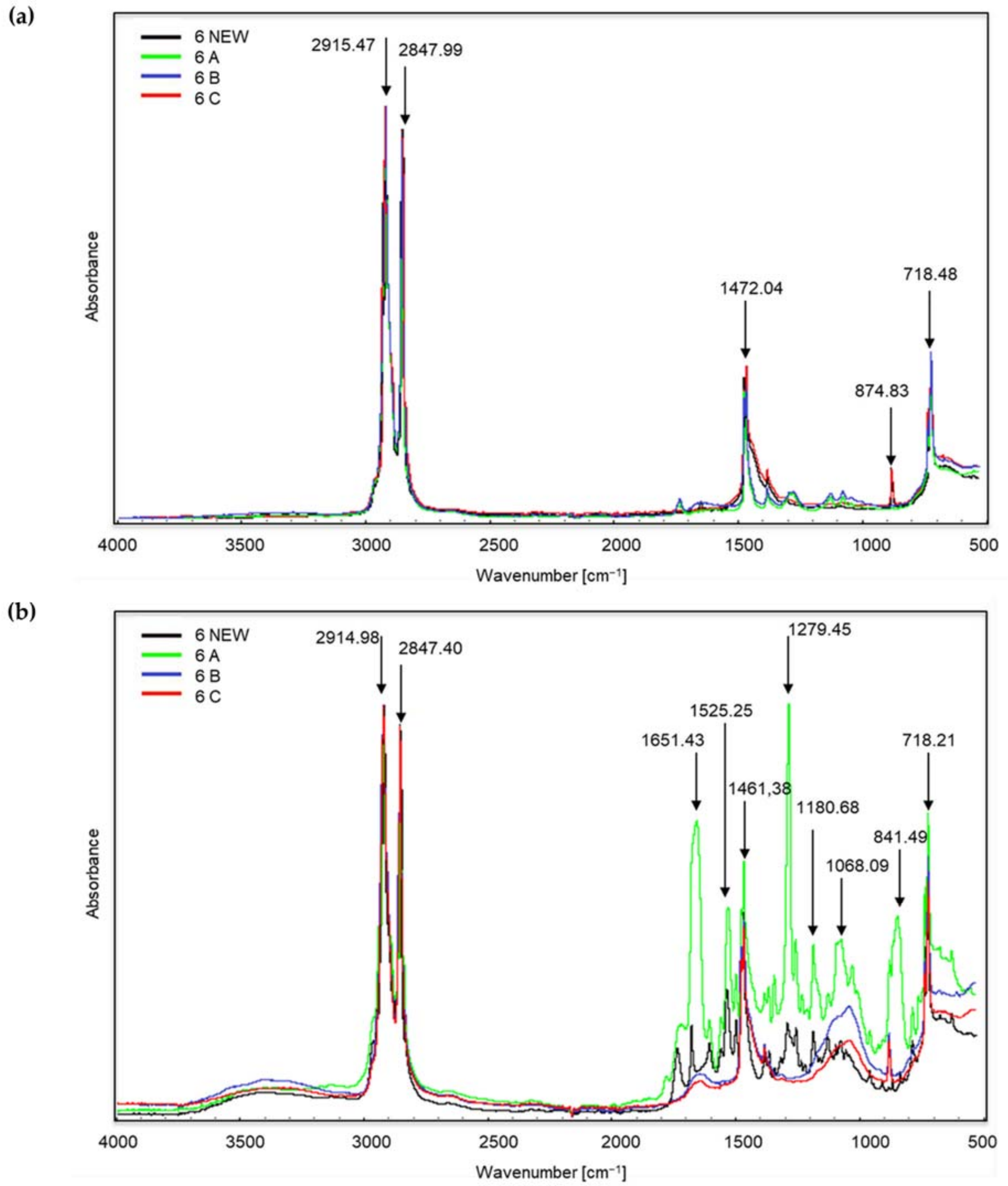

Figure S2. Sample no. 6 spectra: **(a)** spectra generated by the white part of the sample material, **(b)** spectra generated by the coloured part of the sample material.

(a)

(b)

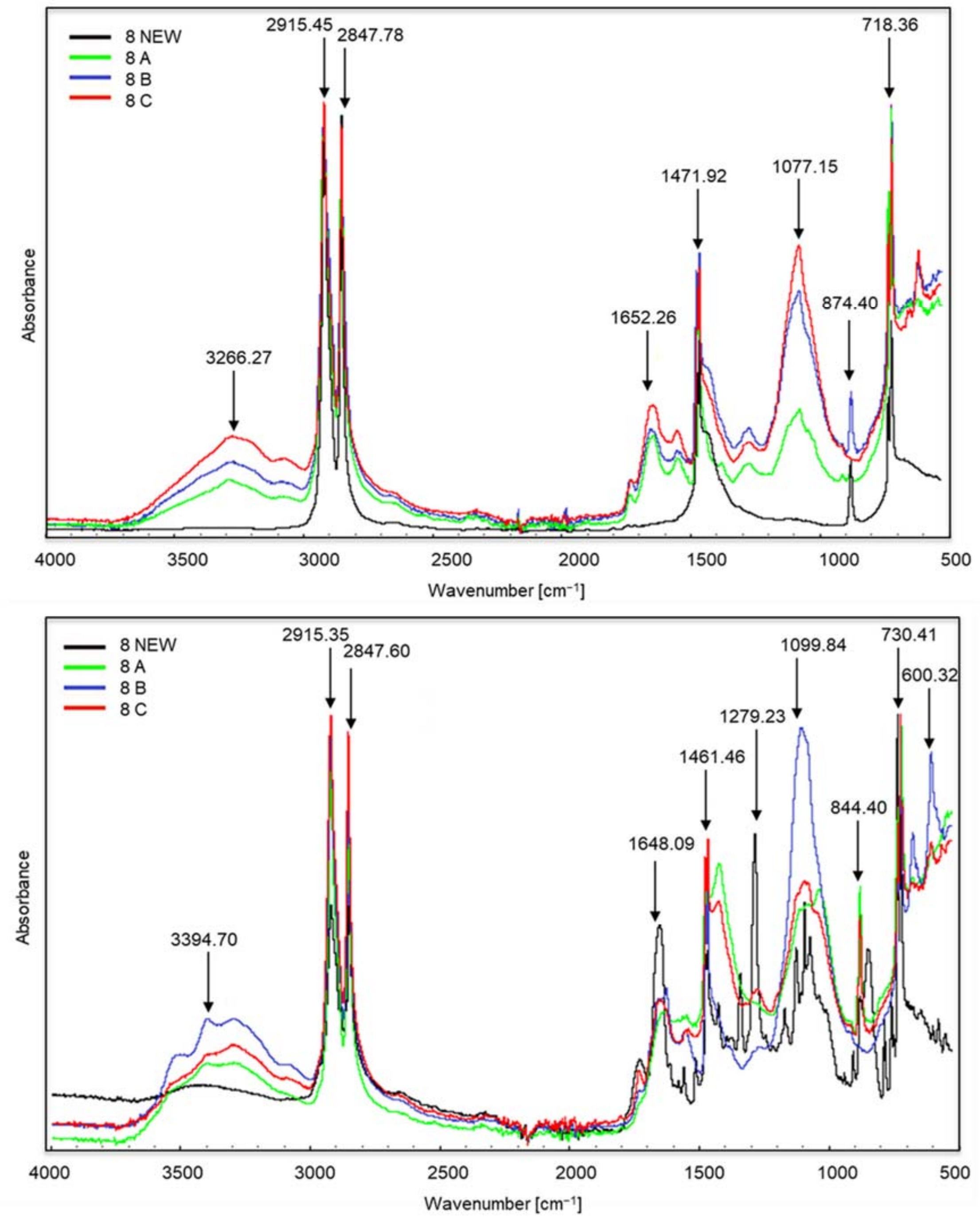

Figure S3. Sample no. 8 spectra: **(a)** spectra generated by the white part of the sample material, **(b)** Scheme 4. Spectra of sample no. 9 (the entire sample was made up of white material).

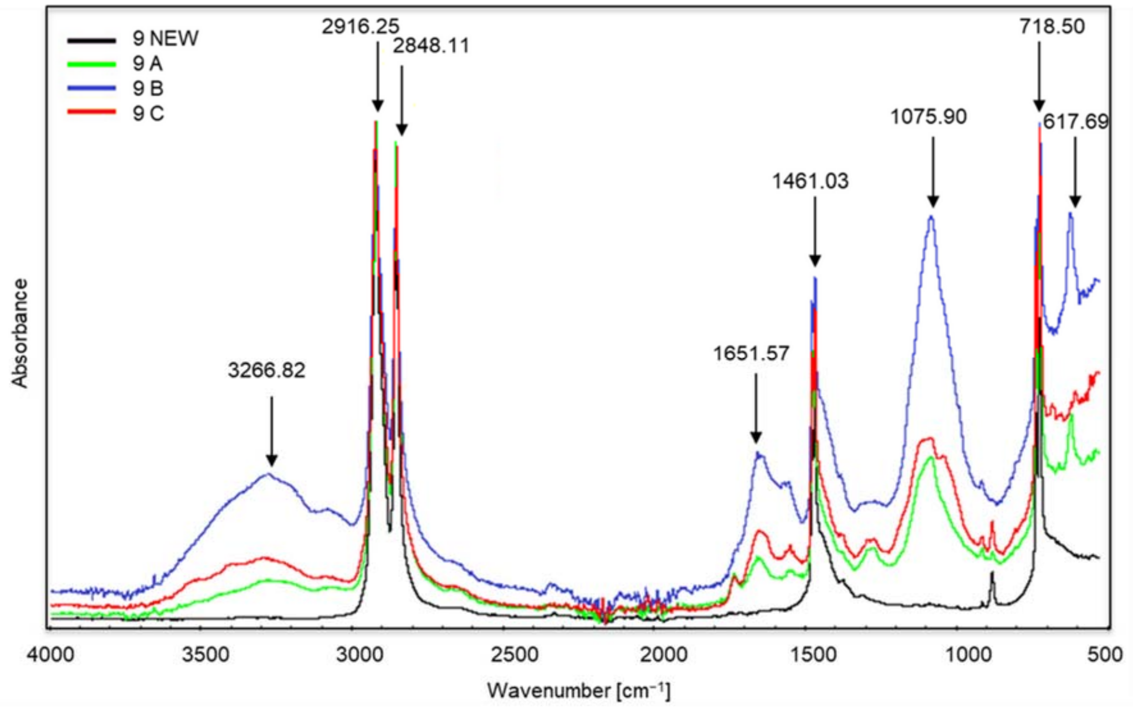

Figure S4. Spectra of sample no. 9 (the entire sample was made up of white material).

(a)

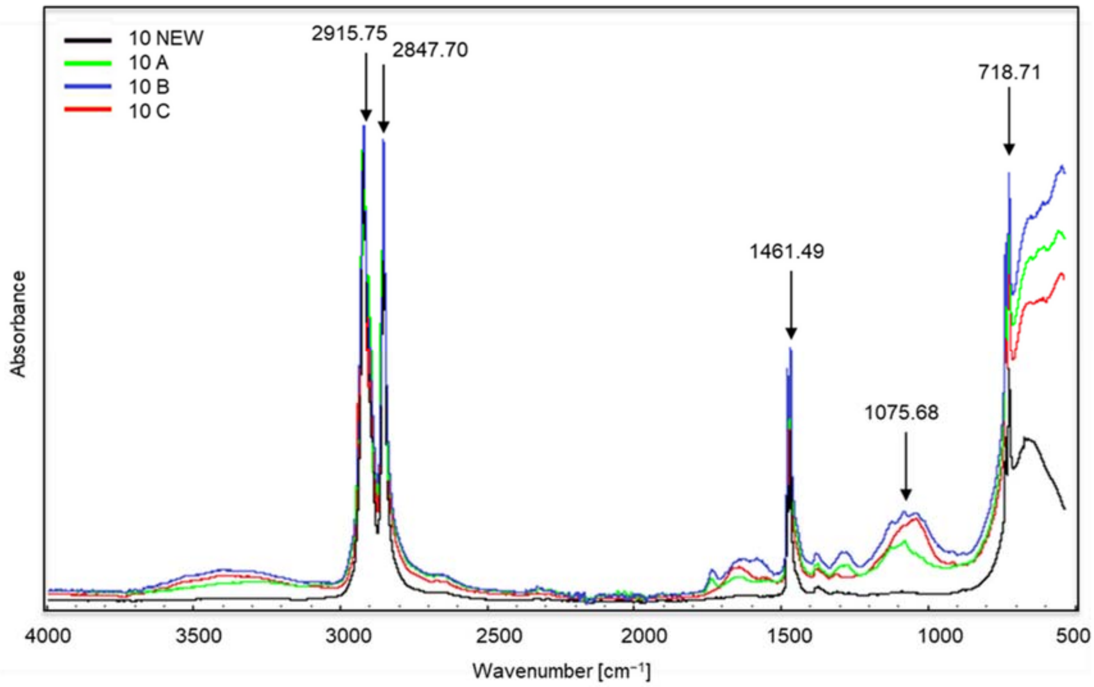

(b)

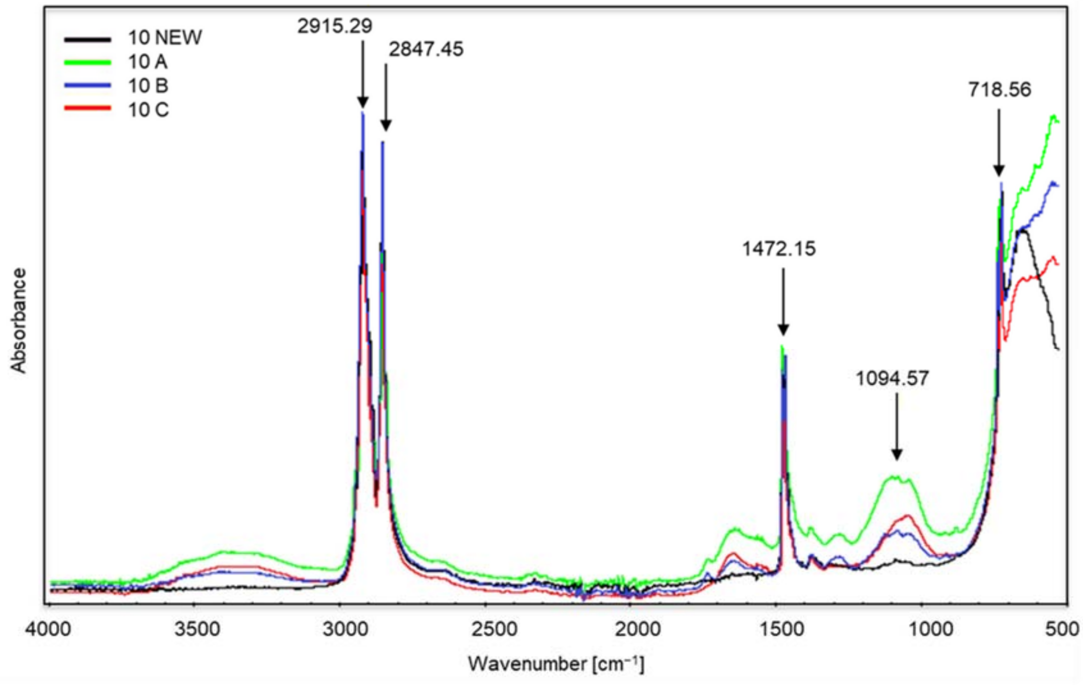

Figure S5. Sample no. 10 spectra: **(a)** spectra generated by the white part of the sample material, **(b)**spectra generated by the coloured part of the sample material.

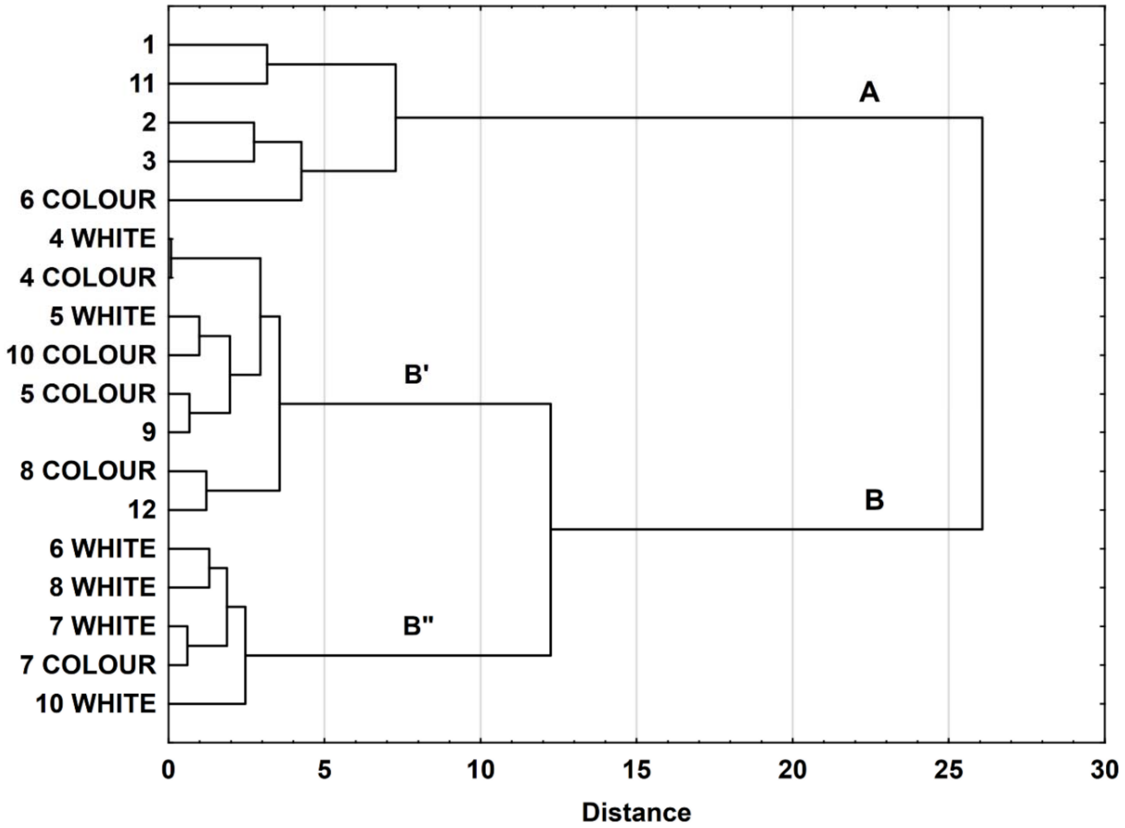

Figure S6. Dendrogram showing the analyzed new samples. Agglomeration was carried out using the Ward method.
